# In vivo optic nerve head mechanical response to intraocular and cerebrospinal fluid pressure: imaging protocol and quantification method

**DOI:** 10.1038/s41598-018-31052-x

**Published:** 2018-08-23

**Authors:** Massimo A. Fazio, Mark E. Clark, Luigi Bruno, Christopher A. Girkin

**Affiliations:** 1 Department of Ophthalmology and Visual Science, School of Medicine, The University of Alabama at Birmingham, 1720 University Blvd., Birmingham, Alabama United States; 2 Department of Biomedical Engineering, School of Engineering, The University of Alabama at Birmingham, 1670 University Blvd., Birmingham, Alabama United States; 3 Department of Mechanical, Energy and Management Engineering, The University of Calabria, Via Pietro Bucci cubo 44C, Arcavacata di Rende, Cosenza Italy

## Abstract

This study presents a quantification method for the assessment of the optic nerve head (ONH) deformations of the living human eye under acute intraocular pressure (IOP) elevation and change of cerebrospinal fluid pressure (CSFP) with body position. One eye from a brain-dead organ donor with open-angle glaucoma was imaged by optical coherence tomography angiography during an acute IOP and CSFP elevation test. Volumetric 3D strain was computed by digital volume correlation. With increase in IOP the shear strain consistently increased in both sitting and supine position (p < 0.001). When CSFP was increased at constant IOP by changing body position, a global reduction in the ONH strain was observed (−0.14% p = 0.0264). Strain in the vasculature was significantly higher than in the structural tissue (+0.90%, p = 0.0002). Retinal nerve fiber layer (RNFL) thickness strongly associated (ρ = −0.847, p = 0.008) with strain in the peripapillary sclera (ppScl) but not in the retina (p = 0.433) and lamina (p = 0.611). These initial results show that: CSFP independently to IOP modulates strain in the human ONH; ppScl strains are greater than strains in lamina and retina; strain in the retinal vasculature was higher than in the structural tissue; In this glaucoma eye, higher ppScl strain associated with lower RNFL thickness.

## Introduction

Primary open angle glaucoma (POAG) remains one of the leading causes of irreversible blindness^1^. The biomechanical response of the optic nerve head (ONH) to changes in IOP varies widely among individuals and is thought to modulate pathologic remodeling of the lamina cribrosa (LC) and sclera that results in axonal injury and eventual retinal ganglion cell death^2^. The driving force behind acute axonal injury and chronic remodeling of the LC in glaucoma is the strain within the LC microenvironment (tissue deformations) which is mediated by two opposing loading mechanical forces: IOP and cerebrospinal fluid pressure (CSFP) which define the translaminar pressure difference (TLPD)^3^.

Computational studies suggest that unlike the oppositional forces from CSFP, there are 2 components of IOP-induced deformation of the ONH: (1) posterior displacement of the LC by the direct effect of IOP, which is counterbalanced by (2) IOP-induced scleral canal expansion, which pulls^4^ the LC anterior within the canal. Thus, the strains experienced within the LC in response to changes in IOP are thought to be modulated by the rigidity and strain in the peripapillary sclera (ppScl)^5,6^. Several computational studies have suggested that the direction and magnitude of LC displacement are strongly related to scleral mechanical behavior^7,8^, with a stiffer sclera resulting in less canal expansion and more posterior deformation of the LC. Theoretically, in contrast to IOP, changes in LC strain seen with variations in CSFP should not be affected by scleral rigidity and should primarily be modulated by LC and ppScl morphology^9^. CSFP exerts a direct effect on the LC in direct opposition to IOP, with lower CSFP causing the LC to displace posteriorly and higher CSFP producing anterior displacement, as is the case with papilledema. Thus, while reduction in CSFP should generate strain in the LC and immediate ppScl, the distribution and magnitude of these strains especially in the ppScl should differ from the strains seen with IOP elevation.

Unfortunately, unlike IOP, non-invasive estimates of CSFP are difficult *in vivo*. CSFP changes with body position, but IOP also changes as well, thus positional changes in the TLPD cannot be precisely estimated in living humans^10^, and thus, the differential effect of CSFP and IOP on LC and ppScl strain cannot be estimated independently. The methods presented in this study allow for the control of IOP while changing body position which affords the ability to estimate and alter CSFP using the extinction point for spontaneous venous pulsation in a living human eye. The purpose of this study is to present the imaging protocol and quantification methods to differentially estimate *in vivo* ONH deformations in response changes in IOP and CSFP within the LC, retina and ppScl using digital volume correlation (DVC) in the cannulated living eye.

## Methods

This study presents new investigation methods evaluated using a novel resource, the Living Eye Project (LEP). The LEP is a collaborative effort between the UAB Department of Ophthalmology and Visual Sciences, the Alabama Organ Center (AOC), and the Alabama Eye Bank (AEB). The AOC developed and opened the Organ Recovery Center at UAB in 2015 as a regional center to provide centralized management for the workup and preparation of donors for organ and tissue transplant. Traditionally, when an organ donor is declared brain dead, transplant surgeons at UAB and AOC staff will travel to hospitals throughout the state and beyond frequently to recover organs from deceased donors. This innovative facility allows for the donors to be moved to the AOC and maintained on life support. This decreases the total time from procurement to transplant and has improved transplant outcomes. LEP also provides a unique platform for research in which examination, imaging, and mechanical ocular testing not achievable in clinical patients can be performed *in vivo*. After the *in situ* testing, the same tissues can be immediately made available for *ex vivo* testing and analyses. Following organ recovery, the ocular tissues are collected by AEB and made available for histomorphometric and histologic study along with cellular and molecular biologic procedures. This approach affords a unique opportunity to correlate *in vivo* ocular biomechanics with *ex vivo* testing. All equipment needed for the described methods for ocular examination/testing and imaging are stored and readily available in the AOC. At the time of organ harvest, the AEB technicians enucleate the eyes in this center and provide them to the investigators. This coordination has provided eyes with unprecedented death-to-preservation times of less than two hours. Data analysis has been performed in Mathematica v.11 (Wolfram Research. Inc.; Champaign, Illinois, USA).

### Donor demographics

**Figure 1:**
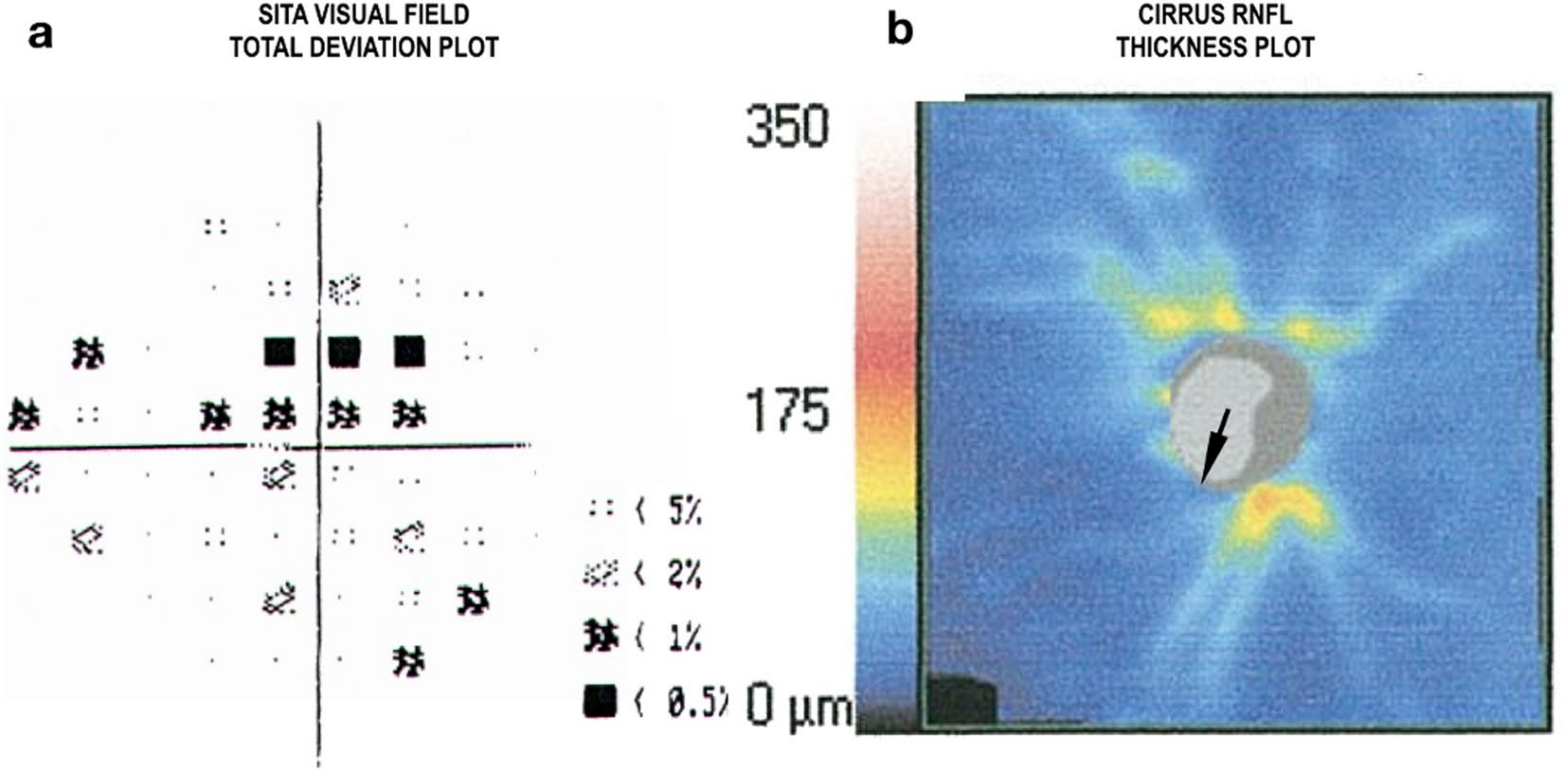
Clinical data of the glaucoma brain-dead organ donor. (**a**) Standard automated perimetry total deviation plot showing a superior arcuate defect and early inferior loss. (**b**) retinal nerve fiber layer thickness map from the patient’s clinical records showing rim notching (small arrow) and inferior > superior thinning.

The right eye of a brain-dead organ donor was tested for this study in the Donor Recovery of the AOC and the University of Alabama at Birmingham (UAB). All the methods and procedures in this study were approved by the University of Alabama at Birmingham’s Institutional Review Board and adhered to the Declaration of Helsinki. Consent for research for the donor included in this report was obtained from the next of kin by a certified AOC coordinator after careful explanation of all procedures. This consent included permission to contact the donor’s treating physicians. The donor in this report was a 51 y/o female of African descent for which we obtained extensive clinical records verifying the donor had chronic open angle glaucoma. Clinical records included the last two clinical chart notes that documented examinations that were performed at approximately 40 months and 4 months prior to death. The initial examination performed by a general ophthalmologist documented open angles on gonioscopy, untreated pressures of 22 in the right eye and 24 in the left eye and a cup-to-disc ratio subjectively estimated as 0.7 with rim thinning in both eyes. The corneal thickness was 492 in the right eye and 496 in the left. The patient underwent and laser trabeculoplasty in both eyes but was lost to follow-up until the final examination 4 months prior to death, which was performed by a glaucoma specialist. This examination documented and confirmed the diagnosis of open-angle glaucoma. The last recorded IOP was 24 mmHg in the right and 16 in the left. The clinician diagnosed progressive optic nerve changes in the right eye and recommended trabeculectomy. The patient refused treatment. The final examination records included the standard automated perimetry (SITA-standard, Humphrey Visual Field Analyzer (Carl Zeiss Meditec; Germany) and spectral Domain OCT (Cirrus OCT, Carl Zeiss Meditec; Germany) obtained by the treating glaucoma specialist which showed a superior arcuate visual field defect corresponding to inferior retinal nerve fiber layer loss and inferior neuroretinal rim thinning OCT (Fig. 1).

### Imaging devices

OCT imaging was performed with a second-generation spectral domain OCT angiography device equipped with a research software (Spectralis OCT-A: Heidelberg Engineering Inc., Germany) and modified to mount the imaging head on a custom counterweighted support arm that allows for six-axis fine manipulation (Spectralis Flex Module, Heidelberg Engineering, Inc.; Germany). In the supine position, a baseline high-resolution 10° cube scan was performed which consists of 512 B-scans of 512 A-scans each with 6 images averaged/B-scan. Axial and lateral resolution was 3.87 µm and 6 µm, respectively, for a total of 512 × 496 × 512 voxels. All scans were performed using enhanced depth imaging mode (EDI). All the OCT images are shown in their original pixel size (not scaled). Proper voxel scaling has been taken into consideration for the computation of the displacement and strain fields.

### IOP control

IOP was measured prior to cannulation with a Tonopen (Reichert Inc., Buffalo, NY) and found to be 12 mmHg. IOP was manometrically controlled by anterior chamber cannulation and by adjusting a saline (PBS) bottle height. The anterior chamber was cannulated with a 27-gauge needle. A digital manometer (XPi, Crystal Engineering; San Luis Obispo, CA) was placed before the needle to monitor inflow pressure. The inflow was controlled by electronically controlled electromagnetic valves (360P012-42 Solenoid Pinch Valve, Neptune Research; Florida). The cornea was lubricated with balanced salt solution and a hard contact lens was fitted on the cornea to prevent cornea dehydration and avoid magnification changes during the follow-up imaging. The lens power was selected to best approximate a neutral spherical refraction based on wet retinoscopy.

### CSFP control

**Figure 2:**
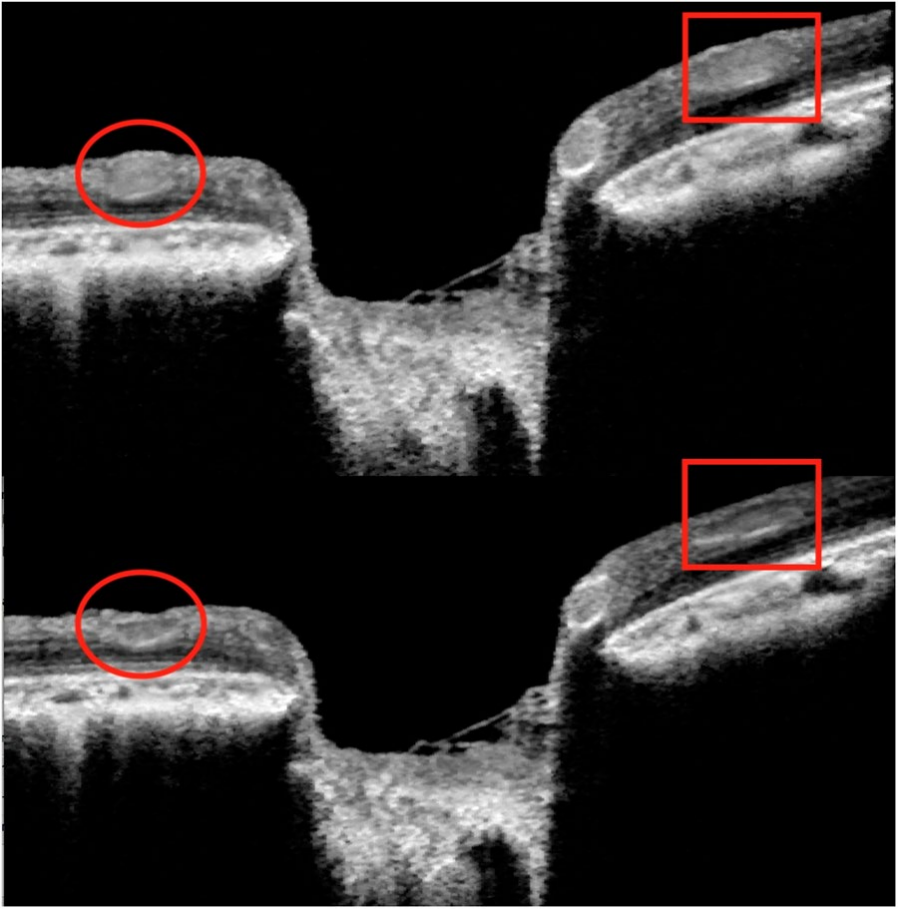
Visualization of the retinal spontaneous venous pulsation. Following manometrically controlled gradual increments in IOP from 5 to 50 mmHg, a subtle variation of the retinal vessel caliber was noticeable at both the confocal scanning laser ophthalmoscope live fundus image and the live vertical central B-scan of the ONH. The frame acquisition rate was approximately 13 Hz. Video recording of the live B-scan showing appearance and cessation of the pulsation induced by a rapid increase in IOP is provided in the supplement materials.

The AOC does not allow direct invasive measurements of CSFP in organ donors. Moreover, ventricular pressure may not represent the CSFP at the level of the ONH^11^. In accordance with the approach used by Golzan *et al*.^12^, we estimated CSFP by measuring the IOP level at which spontaneous venous pulsation (SVP)^13^ was unequivocally visible in both the infrared video image and the OCT live B-scan of the central ONH. At this point, SVP = 0 mmHg (SVP_0_). Given that SVP = r(IOP-CSFP), r = constant, when SVP = 0 then SVP_0_ should equal CSFP in the retrobulbar nerve sheath. Since the SVP measurement error approximates the error due to applanation tonometry, the accurate and precise IOP manipulation afforded by direct cannulation has been demonstrated to provide a reliable estimate of CSFP^13^. This approach has shown a strong correlation with the intracranial Doppler method to measure CSFP and direct intracranial pressure monitoring^13,14^ and is described in brief below. A visual representation of the pulsation and collapse of the peripapillary retinal vessels under an acute IOP increase from 10 to 50 mmHg is provided in Fig. 2. A video recording showing the ONH morphology and collapse of the retinal vessels and SVP of the arteries is provided as supplementary material. While using the funduscopic image and the OCT live B-scan the pulsations can be clearly visualized, the estimate of CSFP using this method requires subjective assessment. While this subjective approach may create some error, in this subject, definite unequivaval pulsations were noted within 2 mmHg of any suggestive change to the pulsatile activity of the vessel.

### Imaging protocol

In the supine position, after cannulation, the IOP was adjusted in 1 mmHg increments until the extinction point, supine SVP_0_, was reached and recorded to estimate CSFP. Imaging was then performed in the seated position with the head stabilized in a custom harness. Preconditioning was performed after cannulation to recover any potential residual compression of the eyeball due to the insertion of the needle. Mechanical state of the eye was pre-conditioned by applying 10 consecutive IOP cycles of increased and decreased pressure from 10 mmHg to 30 mmHg. Once back to 10 mmHg baseline pressure, IOP was incrementally set at 10 and 30 mmHg. A raster scan was acquired at each IOP level after 3 minutes of rest time was allowed after each IOP elevation. The scan at 10 mmHg of IOP and supine position was set as the reference for the follow-up scans, which were acquired with the TruTrack^TM^ eye movement tracking to avoid motion artifact and maximize the communal scanned area across scans. The donor was then moved into a seated position. Following body position change, approximately 10 minutes intercurred before imaging was restarted. Seated SVP_0_ was then determined, and a raster scan was acquired at an IOP of 10, 30, and 50 mmHg. The imaging at 50 mmHg in the supine position was not performed due to the limited available time before the beginning of the organ collection procedure.

### Volumetric 3D strain computation

Volumetric 3D displacement and strain fields were computed by means of a template-matching approach based on digital volume correlation (DaVis v.10, LaVision, Gmbh; Germany). The volumetric 3D strain computation method is a technique extensively used in MRI image analysis and recently applied to analyze deformations of spectral domain optical coherence tomography (SDOCT) volume scans (OCT elastography)^15–17^. General details of the DVC method, firstly applied to OCT images by Girard and colleagues, have been described previously^16,17^. Maximum Shear Strain was used as a metric for mechanical tissue deformations. Areas of low visibility caused by overlaying vasculature or poor OCT signal were excluded from the displacement correlation search. These areas were maximal in the 10 mmHg scan due to the higher blood perfusion, when compared to the 30 and 50 mmHg IOP scans. The OCT scan acquired at 10 mmHg of IOP and at supine position was used as reference volume for the displacement computation. An isotropic kernel of 36 pixels was used for in the correlation analysis. A first iteration of the DVC analysis was used to remove rigid body motion displacement components from the follow-up scans. The rigid body motion was computed relative to the center of mass of each volume by means of polar decomposition separating rotation and deformation components of the vector field. Removing rigid body motion does not affect the strain values.

### Structural and angiography imaging mode

The Spectralis OCT-A, by measuring the movement of light-scattering objects in the scanned volume, provides a non-invasive angiography 3D map of the imaged volume. In contrast to standard OCT, OCT-A analyzes not only the intensity of the reflected light but also the temporal changes in the reflection caused by moving particles in the blood, such as erythrocytes flowing through vessels. These changes in the OCT signal are detected by repeatedly capturing OCT images at each point on the retina to allow for the creation of an image contrast between the perfused vessels (angiography volume) and the static surrounding tissues (structural volume). Structural and vasculature tissue data are in this way intrinsically colocalized. The structural and angiography volumes were used to compute the volumetric deformations and the 3D vasculature anatomy, respectively. The 3D vasculature anatomy was obtained by the reference scan (IOP:10; supine position) as computed by the HEYEX software of the Spectralis (SPX-1710). Only the strain change with imaging modality analysis in the peripapillary Retina region (ppRetina) was analyzed, considering that this region is minimally affected by projection artifacts in comparison to the LC and ppScl in the deep nerve head.

### Data and statistical analysis

Regional variation of the IOP- and CSFP-induced strain was computed for the LC, ppScl, and ppRetina in both the structural and angiography volumes. One thousand voxels were randomly drawn from the LC, ppSlc, ppRetina regions for both the imaging mode volumes (structural and vascular volumes). A sensitivity analysis showed that one thousand sample points per each region was a suitable tradeoff between computational costs and stability of the average regional strain values. A set of 24 equispaced radial scans were extracted from the reference volume (IOP:10;CSFP:24); the 3 regions were manually delineated by an experienced imaging technician using custom 3D delineation software (Multiview^18^); the delineation surfaces were then interpolated and realigned to the reference volume. Variations of the sectorial mean strain in the LC, ppScl, and ppRetina as a function of IOP and CSFP were analyzed by linear mixed-effect models (NLME package - R - Foundation for Statistical Computing; Vienna, Austria). An exponential spatial correlation structure accounted for the autocorrelation of the residuals within each scan volume as a function of their angular distance.

### Strain vs. RNFL thickness correlation

Sampling points from the LC, ppSlc, and ppRetina were gathered in 8 sectors oriented according to the TSNIT convention. From the circular scan of the right eye, the RNFL sectorial variation was interpolated with a continuous function. This function associated strain values of the sampling points to the RNFL thickness value depending on their angular position. The strain values were computed for an IOP elevation of 20 mmHg (IOP = 30 vs. IOP = 10 mmHg scan). A Pearson R test analyzed the correlation between average sectorial strain and RNFL thickness.

## Results

**Figure 3:**
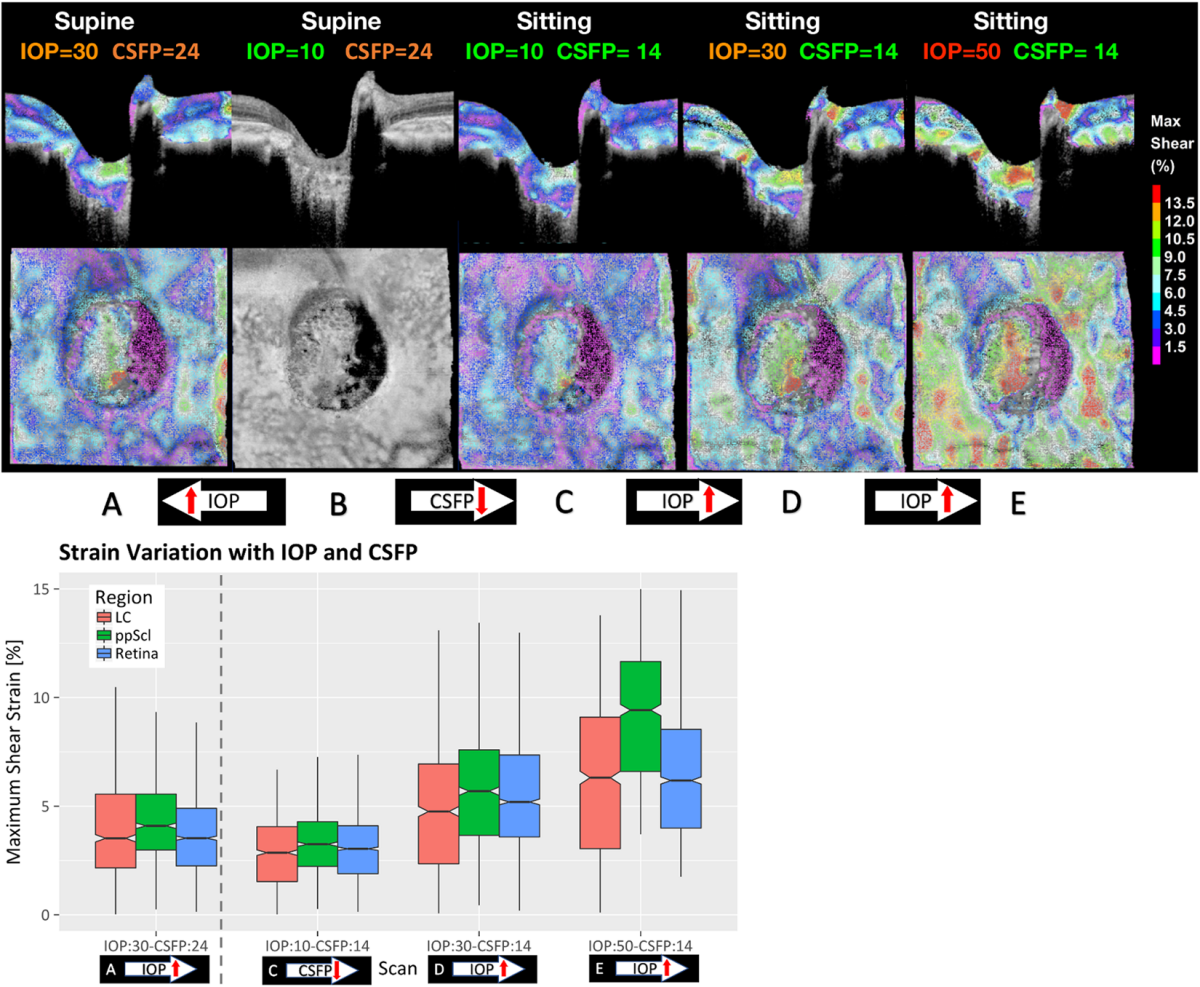
(**u**) 3D shear strain maps in both sagittal and transverse views of all the scans. The scan at baseline IOP and supine position was used as reference volume for the imaging and strain computation. Comparing (B->C vs. B->A): lowering of CSFP alone (B->C) induces LC strain similar to elevation in IOP (B->A): comparing (B->D vs. B->A): the same IOP increase, there is higher strain increase in the scan at lower CSFP (D vs. A). (**l**) Box-whisker plots of the regional strain distribution for the follow-up scans.

**Figure 4:**
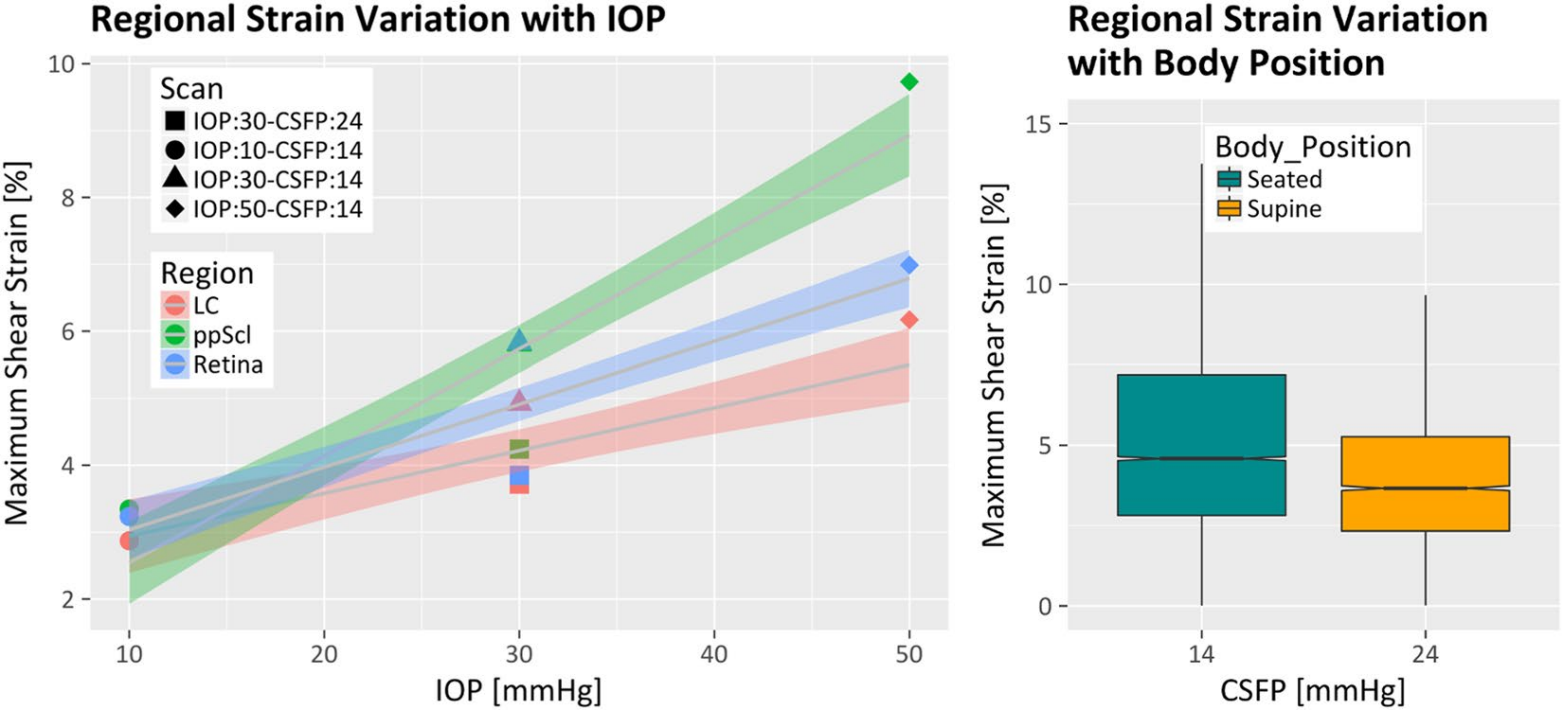
(**l**) Regression line and confidence intervals of the change in Maximum Shear Strain with IOP for the lamina cribrosa (LC), peripapillary (ppScl), and peripapillary retina regions. Each marker represents the average strain difference between each follow-up scan against the reference scan (IOP:10; CSFP:24). Strain was consistently lower for each ONH region in the scan at supine position. **(r**) Strain change associated with a variation in CSFP at constant IOP (30 mmHg). To a CSFP increase the average strain decrease of −0.14%/mmHg (p < 0.0264), which was comparable in magnitude to the strain increase associated with an increase in IOP.

**Figure 5:**
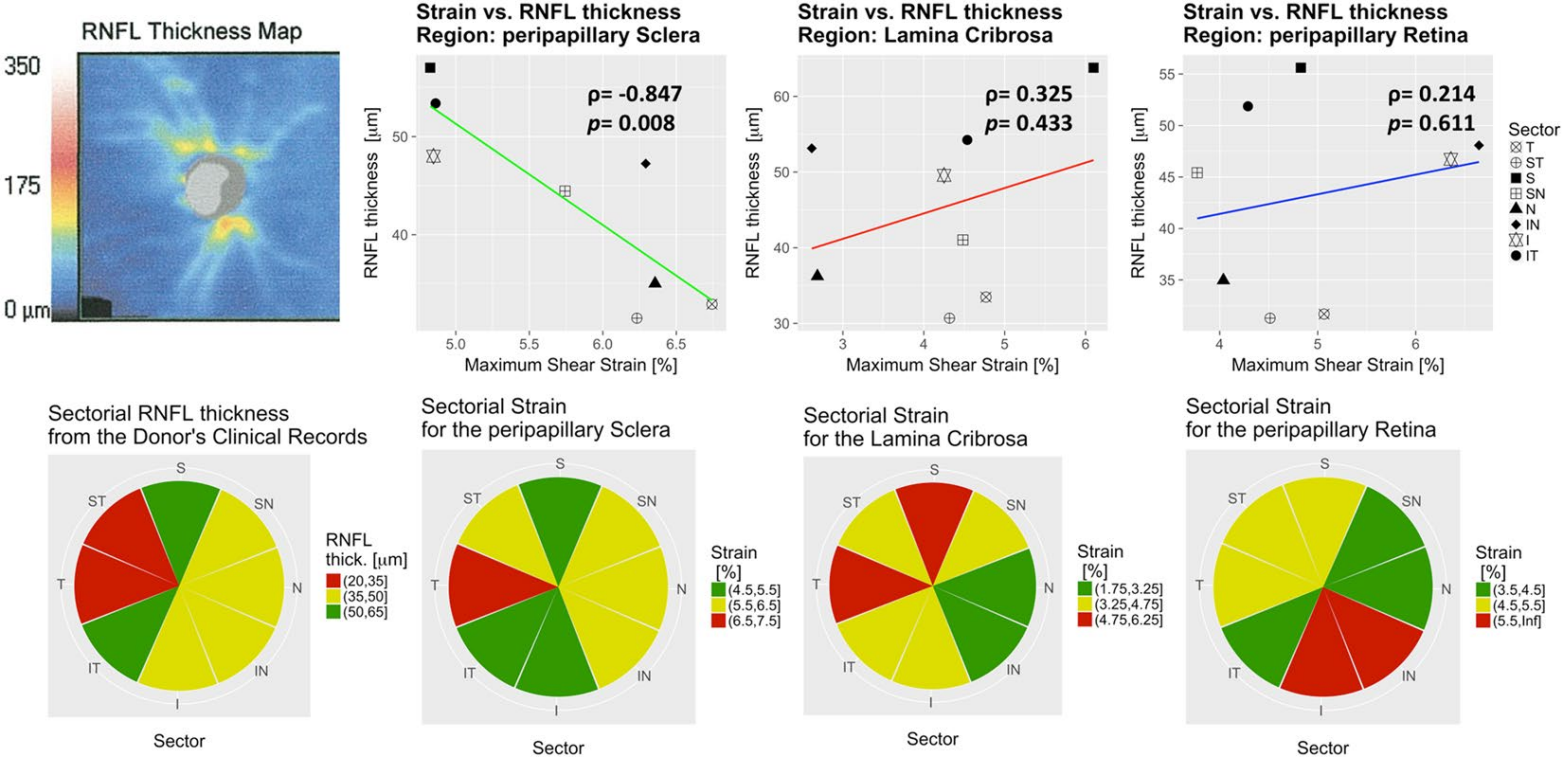
Strain vs. retinal nerve fiber layer (RNFL) thickness correlation. From the RNFL thickness map obtained donor’s clinical records (column 1, row 1), the RNFL map of the OD eye was computed in a conventional 8-sector clock-hour map (r2, c1). Pearson’s correlation of the RNFL thickness as a function of the maximum shear strain showed a strong correlation for the region of the peripapillary sclera (ρ = −0.847, p-value = 0.008; c2) while neither the strain in the lamina cribrosa or peripapillary retina showed a correlation with RNFL thickness (p-value = 0.433 and p-value = 0.611; c3 and c4).

**Figure 6:**
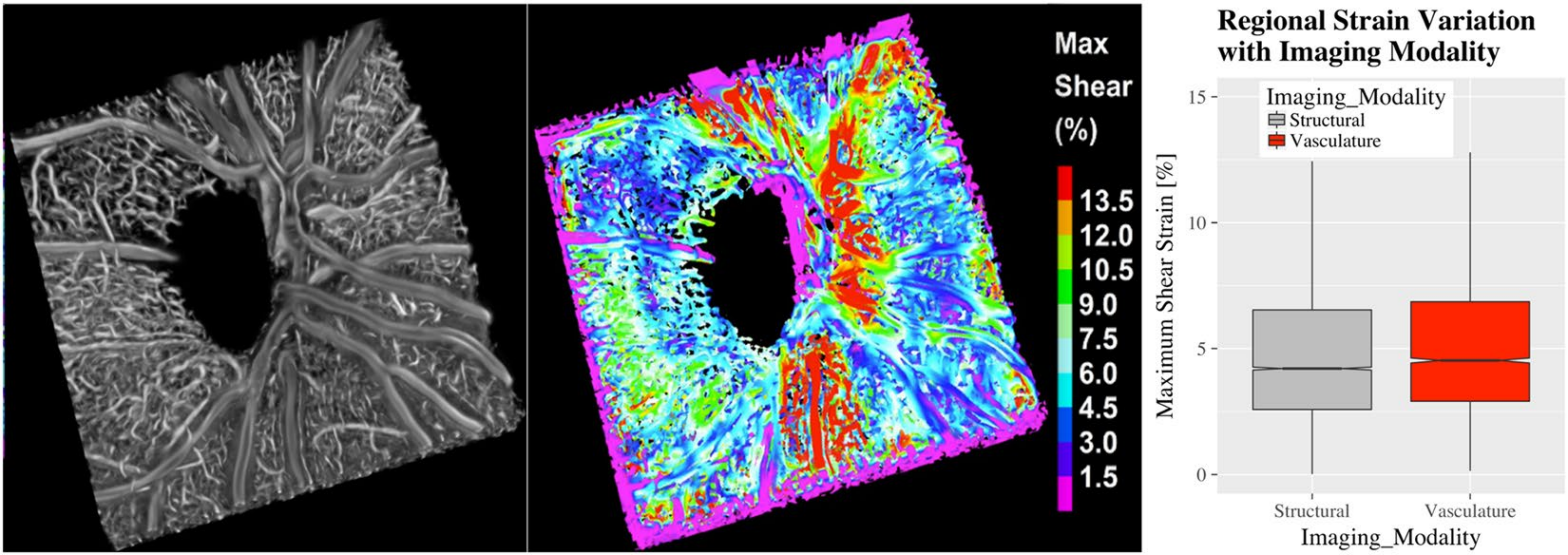
(**l**) Colocalization of SDOCT vasculature with Strain: (left) 3D map of the vasculature in the peripapillary retina (ppRetina) with co-localized to the strain (right) experienced by these tissues with an IOP change from 10 to 30 mmHg. (**r**) Distribution of strain in the ppRetina as it varied with imaging modality showing that strain localized in the vasculature was higher than in the structural tissue (0.90%, p = 0.0002).

Maximum shear strain in the ONH of a sagittal and frontal section of the volumetric 3D strain map at each IOP and CFSP level is shown in Fig. 3u. Average strain distribution changes in each region for each follow-up scan are shown in the box-whisker plot in Fig. 3l. Outcomes of the multivariate regression analyses are reported in Table 1. Regional variation of the maximum shear strain change with IOP is shown in Fig. 4l; box-whisker plot of the strain variation with CSFP is shown in Fig. 4r. Correlation between RNFL thickness and strain is shown in Fig. 5. Colocalization of the strain in the vasculature of the retina, and variation of the strain in the ppRetina is shown in Fig. 6l; distribution of strain for the two imaging modalities (structural vs. vasculature) for the ppRetina region is shown in box-whisker plot of Fig. 6r.

**Table 1:** Multi-variate Regression Model of the Regional and Sectorial Strain Variation with IOP, CSFP, and Imaging Modality.

	Parameter	Unit	Estimates	SE	p-value
IOP	LC	%/mmHg	0.06	**0.01**	**<0.001**
	ppScl	%/mmHg	0.16	**0.01**	**<0.001**
	ppRetina	%/mmHg	0.09	**0.01**	**<0.001**
CSFP	Global	%/mmHg	−0.14	0.02	**0.0264**
Imaging Modality	Vasculature	Δ%	0.90	0.23	**0.0002**
Regional Variation - IOP = 10 mmHg	LC	%	3.36	0.42	0
	ppScl	Δ%	−0.43	0.47	0.3546
	ppRetina	Δ%	−0.37	0.43	0.3987
Regional Variation - IOP = 30 mmHg	LC	%	4.17	0.33	**<0.001**
	ppScl	Δ%	1.44	0.31	**<0.001**
	ppRetina	Δ%	0.22	0.31	0.4665
Regional Variation - IOP = 50 mmHg	LC	%	5.72	0.45	**<0.001**
	ppScl	Δ%	3.18	0.53	**<0.001**
	ppRetina	Δ%	0.51	0.53	0.3389
Sectorial Variation	ST	Δ%	−0.59	0.39	0.1359
	S	Δ%	−0.30	0.41	0.4767
	SN	Δ%	−1.05	0.39	**0.0081**
	N	Δ%	−1.22	0.40	**0.0027**
	IN	Δ%	−0.24	0.41	0.558
	I	Δ%	−0.14	0.39	0.7208
	IT	Δ%	−0.92	0.39	**0.0202**

The inferior sector was set as the intercept. ppScl = peripapillary sclera; ppRetina = peripapillary retina; IOP = intraocular pressure; CSFP = cerebrospinal fluid pressure.

### Effect of IOP

An increase in IOP resulted in a significant rise of the average strain in the LC (0.06%/mmHg, p < 0.001), ppScl (0.16%/mmHg, p < 0.001) and ppRetina (0.09%/mmHg, p < 0.001; (see Table 1, *IOP*). Mean regression and 95% confidence intervals of the strain change with IOP as predicted by the regression model are shown in Fig. 4l. Regional variations of strain significantly varied with increasing IOP (see Table 1, *Sectorial variation*). At baseline IOP (10 mmHg) and low CSFP (14 mmHg) the regional strain was non-significantly different across the 3 regions of the ONH (LC, ppRetina, and ppScl; Table 1, *Regional Variation - IOP* = *10 mmHg*). Strain in the ppScl at 30 and 50 mmHg of IOP was significantly higher (p < 0.001) than in the LC and ppRetina.” (Table 1, *Regional Variation - IOP* = *30 mmHg* and *50 mmHg*). Strain in the ppRetina was not found to be significantly different from the average strain in the LC at any level of IOP (p = 0.3987, p = 0.4665, p = 0.3389; Table 1).

### Effect of CSFP

With a change in body position from seated to supine at constant IOP, CSFP increased from 14 mmHg to 24 mmHg as estimated by SVP_0_. Associated with this variation in CSFP, a significant decrease in the average global strain was observed (−0.14%/mmHg, p = 0.0264, Table 1), as shown in the box-whisker plot in Fig. 4r. Regional distributions of the strain change with CSFP are shown in Fig. 3l.

### Sectorial strain vs. RNFL thickness correlation

Sectorial analysis showed that the strain in the temporal sector (used as a reference) was higher than the nasal (1.22%, p = 0.0027), superonasal (1.05%, p = 0.0081), and inferotemporal sector (0.92%, p = 0.0202; Table 1, *Sectorial Variation*). The average sectorial maximum shear strain showed a strong negative correlation with the RNFL thickness in the ppScl (ρ = −0.847, p = 0.0008; Fig. 5 column 2). A correlation with RNFL thickness was not present for the strain in the LC (p = 0.433) and ppRetina (p = 0.611) regions (Fig. 5, column 3 and column 4). Sectorial distribution of the RNFL thickness and corresponding average strain is shown for each region in the second row of Fig. 5.

### Effect of the imaging modality

The strain localized in the vasculature tissue of the ppRetina was quantified and compared to the average strain in the structural tissue of the same region. A visualization of this computation is shown in Fig. 6l where the volumetric strain map (left 3D view) is colocalized to the 3D anatomy of the vasculature (right 3D view). On average, strain in the vasculature tissue was 0.9% higher (p = 0.0002; Table 1) than the in the non-vasculature tissue (structural tissue). Comparison of the strain distribution in the structural and vasculature volumes for the ppRetina is shown in Fig. 6r.

## Discussion

We developed an imaging and quantification method to resolve ONH mechanical response to changes in IOP and CSFP in the living human eye of a brain-dead organ donor. This approach provides an estimation of the biomechanical response of the ONH to changes in IOP and CSFP independently for the first time in a living human eye. In addition, the computational methods employed allowed for the calculation of strain within regions of the SDOCT volume representing the visible lamina cribrosa, sclera, and retina separately, and within distinct vascular (time-varying) and structural (stationary) constituent tissues. Our initial results provide several noticeable observations: (1) Elevation of IOP and reduction in CSFP resulted in directionally similar deformation of the ppScl, LC, and ppRetina (Table 1 and Fig. 3); (2) ppScl strains were greater than strains in LC and retina, and this difference increased with increasing IOP (Table 1, Figs 4a and 3b); (3) strains within the retina were the greatest in magnitude in the vasculature tissue compared with the structural tissue (Table 1; Fig. 6).

This tested eye with documented open-angle glaucoma showed significant RNFL thinning on the supero and inferotemporal sectors (Figs 1 and 5, column 1 row 1). High strains were observed in these sectors of the LC (Fig. 5, column 3). Higher strain strongly correlated to lower RNFL thickness, in the ppScl (Fig. 5; row 1, columns 2, 3, and 4).

Thanks to the dual imaging modality offered by OCT angiography, we could colocalize volumetric strain data to vasculature 3D morphology. This unique feature of our quantification approach allows for distinguishing the contribution of vasculature deformations to the overall strain in the ONH. Differentiating strain between vascular and non-vascular components of the ONH should increase the accuracy of the computed strain of the load-bearing collagenous tissue of the ONH which is thought to drive glaucomatous remodeling of these tissues.

The ONH consists of load-bearing connective tissues (LC and ppScl) and neurovascular tissue. The LC provides support to the retinal ganglion cell (RGC) axons as they pass from the high-pressure environment in the eye to the lower pressure region in the retrobulbar cerebrospinal space^19^. To protect the RGCs in this anatomic region, the human LC has evolved into a complex 3D network of flexible beams of connective tissue nourished by a capillary bed arising from the short posterior ciliary arteries penetrating the immediate ppScl. This intra-scleral and intra-laminar vasculature is unique in that it is encased in collagenous load-bearing connective tissue^20–22^. Thus, the vascular, neural and cellular components of the LC are inseparably intertwined with its mechanical behavior. A biomechanical model has been proposed to incorporate these concepts^23^. This model proposes that IOP/CSFP-related stress (force/cross-sectional area) and strain (local deformation of tissues) play an essential and causative role in the pathophysiology of the changes seen in all of the tissues within the ONH and its blood supply. Regardless of the primary insult in glaucomatous injury, stress and resultant local deformations (strain) of the laminar connective tissues are key elements in this model and are dependent on the trans-lamina pressure difference (TLPD = IOP − CSFP), the 3D architecture of the ONH^7,19,23–36^ and the mechanical stiffness of the sclera, which are all interrelated^37–40^.

Several studies have suggested that a change in TLPD is potentially a greater risk factor for glaucoma than elevated IOP alone^41–48^. Evidence indicates that CSFP is lower in patients with POAG and normal tension glaucoma (NTG) when compared to controls^41–44^. *Ex vivo* studies in porcine eyes have demonstrated increase LC strain from elevation of nerve sheath pressure^48^. Chronic lowering of CSFP in a primate resulted in RNFL thinning without the connective tissues changes that are typical in the high-IOP model^45^. In rats with directly controlled IOP and ICP (intracranial pressure), retinal blood flow in the older eye was more resistant to IOP elevation compared with its younger counterpart, especially at low and mild ICP elevations^49^. This suggests that changes in the TLPD can induce strain in the LC and may affect the ONH differently than elevation of IOP alone. However, in this intial case study, we did not find the differential effect of IOP and CSFP on the regional average strain to be significant (CSFP-Region interaction term showed a p = 0.39). Lastly, a recent study on primates highlighted how the *in vivo* assessment of the interaction between IOP and CSFP is crucial as many different IOP and CSFP combinations can result in the same TLPD^50^. Thus, TLPD more completely represents the loading force experienced by the LC and ppScl than IOP alone.

Unfortunately, unlike IOP, non-invasive estimates of CSFP are difficult *in vivo*. CSFP changes with body position, but IOP also changes as well, thus positional changes in the TLPD cannot be accurately estimated in living humans^51–53^. The methods developed for this study, conducted using eyes from brain-dead organ donors, allow for controlling IOP while changing body position due to the ability to manometrically control IOP via anterior chamber cannulation. This affords the ability to estimate and alter CSFP using the extinction point for spontaneous venous pulsation^12^. The results of regional strain seen in this study suggest that deformations in the LC and ppScl with elevation of CSFP are similar in magnitude but opposite in direction to elevation in IOP.

Several limitations of the proposed method have to be kept in mind when interpreting these results. The mathematical formulation of the DVC method creates imprecisions in the assessment of the strain within the vasculature tissue. The operation of convolution performed by the correlation analysis imposes smoothing of the deformations that is proportional to the correlation kernel size. This factor may affect the estimated magnitude of the variation of the strain with imaging modality (vasculature vs. structural). The convolution operated by the DVC method also prevents a proper separation of the strain in the LC, sclera, and retina because of their spatial contiguousness. In particular, strain in the ppScl may be influenced by the deformations of the more compressible choroid. These potential effects of the DVC convolution on the ONH strain characterization warrant dedicated investigations as these methods are further developed to define potentially mechanistically-relevant biomarkers for glaucoma.

Change of body position, needed to modulate CSFP, required a realignment of the OCT and the donor’s eye. Similar to clinal practice, some realignment distortions should be expected to be introduced by any realignment procedure, which would potentially lead to artifacts in the strain computation. We mitigated realignment distortions by constraining head motion of the donor by means of a custom-made restrainer. Since the inline digital manometer was external to the eye, the absolute IOP measurement may be affected. However, the manometer was maintained at a similar relative height to the eye with changes in body position. Blood pressure was monitored by the sustained life support equipment and was fairly constant, but small oscillations during the imaging acquisition session cannot be excluded. Considering our results are based on analyzing differential changes of the strain with changes in IOP and body position, we expect for these limitations not to significantly affect the proposed results. We have recently modified the IOP measurement procedure, which now relies on a piezoelectric sensor (Pressure Catheter SPR-671NR; ADInstruments, Colorado Springs, CO) directly inserted into the anterior chamber.

CSFP was non-invasively estimated by assessing the onset of the central vein spontaneous venous pulsations and not by direct cannulation of the brain lateral ventricle (ICP). However, this approach affords the estimate of CSFP specifically within the retrolaminar compartment which may differ from ICP in the lateral ventricle. ICP refers to pressure within the cranial vault, CSFP represents pressures throughout the central neuroaxial system, and retrolaminar pressure - which ultimately affects LC biomechanics – may also differ from ICP^54^. While the CSFP in our study is estimated and not measured directly, ventricular ICP and retrolaminar CSFP pressure are related but not equivalent^55^. Moreover, glaucomatous changes of the lamina cribrosa could potentially increase the trans-lamina cribrosa outflow resistance so that the central retinal vein pressure is no longer directly correlated with the orbital CSFP^56^. Lastly, CSFP was defined using an approach that relies on subjective assessment of venous pulsation. While this is assisted with visualization of the OCT video image and simultaneous CSLO imaging, there is likely some error within this assessment as discussed in the methods above. However, prior work has demonstrated that this method can approximate ICP determined by invasive measurements with a measurement error that approximates the error due to applanation tonometry^13^. Thus, the accurate and precise IOP manipulation afforded by direct cannulation may provide a more reliable estimate of CSFP. Moreover, positional change in CSFP is clearly evident and resulted in deformation (strain) within the ONH. Future studies evaluating this approach in donors that have ICP monitors in place for clinical reasons would be helpful in determining the correlation between retrolaminar CSFP measured using this approach and ICP.

IOP- and CSFP-induced strain of the ONH has been shown to follow a non-linear correlation^50^. The array of four IOP and four ICP independent measurements performed by Wang *et al*.^50^ is an ideal imaging protocol for analyzing the non-linearity of this correlation. Unfortunately, the imaging time available in the AOC for OCT imaging is too limited for the implementation of a similar protocol.

The experimental version of the Heidelberg HEYEX^TM^ software (SPX-1710) does not provide a normative database for the quantification and comparison of the RNFL thickness. For such reason, we rather performed the evaluation of the retinal thickness based on the clinical records of the donor which were acquired by a Cirrus OCT. The Cirrus scan was obtained within 4 months of death. It is highly improbable that significant change occurred in a chronic disease like POAG. The results presented in this study were based on only a single test-case and are meant to present the imaging protocol and the quantification method for analyzing the ONH biomechanical response to IOP and CSFP *in-vivo*. Due to the limited nature of a case report, none of the results presented herein should be considered to be representative of the average biomechanical response of the ONH to IOP and CSFP of the population. A larger group is needed to address such question.

Cause of death can possibly alter the ONH biomechanics. This potential confounder should be taken into consideration when this method is applied to a larger population of donors.

Lastly, while this study demonstrates a computation method employed using a novel platform in brain-dead organ donor eyes to obtain ONH strain measurement under precise manometric control, development of this approach requires expansion to a larger group of subjects to define associations with variations in ONH strain with aging and disease. The availability of these donor tissues for *ex vivo* testing affords the ability to correlate variation in experientially measured *in vivo* ONH strain with *ex vivo* measurements of scleral strain along with an analysis of the cellular and molecular characteristics of the load bearing connective tissues of the ONH.

In conclusion, we presented an imaging protocol and quantification method to investigate ONH mechanical response to changes in IOP and CSFP for the first time in the living human eye. Moreover, this methods affords the opportunity to define strain within specific ONH regions (LC, ppScl, and ppRetina) and compartments (vascular vs. structural) that likey play differing but mechanistically relevant roles in the pathogenesis of glaucomatous optic neuropathy within the framework of the biomechanical model of the disease. Thus, the capability to quantify regional optic nerve, retina, and sclera strain in the living human eye will not only enhance the understanding of the relationships between variations in tissue strain and vulnerability to IOP related ONH injury but may also provide mechanistically relevant structural biomarkers to predict and detect progression of this blinding disease.

## Electronic supplementary material

Supplementary Information

## Data Availability

The datasets generated for the current study are available from the corresponding author on reasonable request.
